# The shape of human squalene epoxidase expands the arsenal against cancer

**DOI:** 10.1038/s41467-019-08866-y

**Published:** 2019-02-21

**Authors:** Andrew J. Brown, Ngee Kiat Chua, Nieng Yan

**Affiliations:** 1 School of Biotechnology and Biomolecular Sciences, University of New South Wales, Sydney, NSW 2052 Australia; 2 Department of Molecular Biology, Princeton, NJ 08544-1014 USA

## Abstract

Squalene epoxidase (also known as squalene monooxygenase, EC 1.14.99.7) is a key rate-limiting enzyme in cholesterol biosynthesis. Anil Padyana and colleagues report the long awaited structure of human squalene epoxidase (SQLE). They solved the crystal structure of the catalytic domain of human SQLE alone and in complex with two similar pharmacological inhibitors and elucidate their mechanism of action. SQLE is the target of fungicides and of increasing interest in human health and disease, particularly as a new anti-cancer target. Indeed, in a companion paper, Christopher Mahoney and colleagues performed an inhibitor screen with cancer cell lines and identified SQLE as an unique vulnerability in a subset of neuroendocrine tumours, where SQLE inhibition caused a toxic accumulation of the substrate squalene. The SQLE structure will facilitate the development of improved inhibitors. Here, we comment on these two studies in the wider context of the field and discuss possible future directions.

## Background

3-hydroxy-3-methylglutaryl-CoA reductase (HMGCR) catalyzes the initial rate-limiting step during cholesterol biosynthesis and is the target of the highly successful statin class of cholesterol-lowering drugs. However, relatively little is known about the 20 or so other enzymes involved in cholesterol synthesis^1^. A case in point is SQLE, which catalyzes a second rate-limiting step downstream of HMGCR and has been relatively neglected until now.

And yet this enzyme is becoming a hot topic. The yeast homologue ERG1 is a target of antifungals like terbinafine. The human enzyme has a long history of being a potential cholesterol-lowering target, and natural products in common foods and beverages, such as garlic, red wine and green tea, may lower blood cholesterol levels by inhibiting SQLE^2^. More recently, human SQLE has been gaining prominence as a bona fide oncogene and target in cancer therapy^3–5^.

**Fig. 1:**
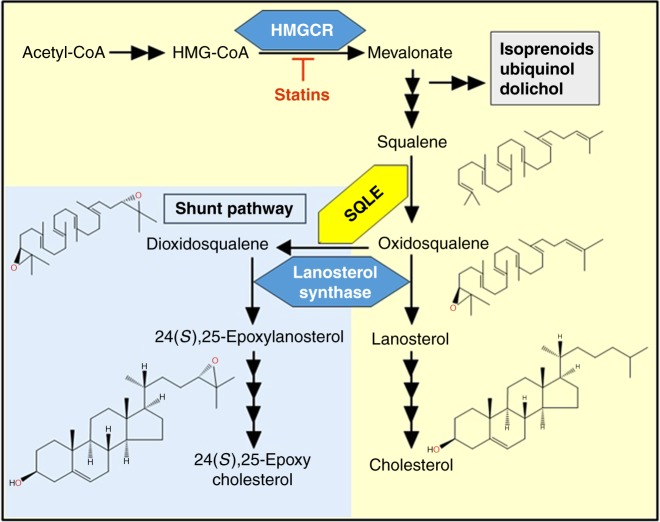
Simplified scheme of the cholesterol biosynthesis pathway featuring the shunt pathway. In the shunt pathway SQLE acts a second time to lead to the production of a potent oxysterol regulator, 24(*S*),25-epoxycholesterol. The oxygen atoms from the epoxidation are shown in red

Like most cholesterogenic enzymes, SQLE is located in the endoplasmic reticulum, and perhaps also on lipid droplets. The enzyme is conserved across eukaryotes, with 45% sequence homology between human SQLE and yeast ERG1. SQLE catalyzes the first oxygenation step in cholesterol synthesis, introducing an epoxide group into the isoprenoid squalene to form 2,3(*S*)-oxidosqualene (Fig. 1). This epoxidation is required before the isoprenoid can be folded into the characteristic steroidal four-fused ring structure, through some remarkable molecular origami catalyzed by the subsequent enzyme, lanosterol synthase.

The molecular interactions of the SQLE enzyme-substrate complex must be exquisitely precise to rigidly control the regio- and stereochemistry of the epoxidation reaction^6^. The resulting oxygen atom becomes the signature hydroxyl group of cholesterol. The reaction requires molecular oxygen, FAD, NADPH, and an electron transfer partner including NADPH-cytochrome P450 reductase^7^. Substrate delivery to the active site of SQLE requires anionic phospholipids and a lipid transfer protein, supernatant protein factor (SPF)^8^.

**Fig. 2:**
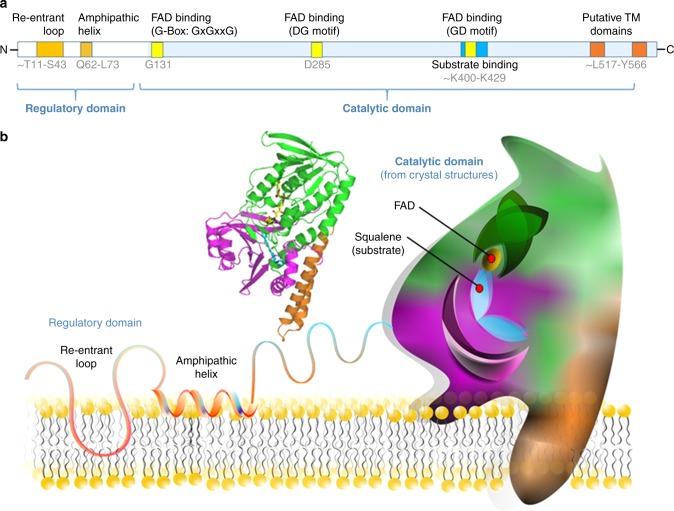
SQLE structure. **a** Linear view of SQLE protein with known structural features. **b** Structure of the catalytic domain of human SQLE^10^. The FAD binding domain is shown in green, the substrate-binding domain in magenta, and the C-terminal membrane-associated helical domain is coloured orange. FAD (yellow) and the inhibitor NB-598 (blue) are shown in stick representation. Schematic model of SQLE partially embedded in the endoplasmic reticulum membrane including a representation of the N-terminal domain based on our previous work^11^

Work over the past two decades has characterized several key structural features of SQLE. The catalytic domain (Fig. 2a) contains the FAD binding motifs (G-Box, GD- and DG-motifs) that are characteristic for flavin monooxygenases. The DG-fingerprint also serves to recognize the NADPH cofactor and is nestled within a patch of residues identified as the substrate-binding site from photoaffinity labelling experiments in recombinant rat SQLE^6, 9^.

Although substrate requirements, cofactors, and inhibitors have been investigated for SQLEs of various origins, no structural model has been available so far. Moreover, the domains responsible for enzymatic activity and inhibitor interactions are not well understood. Therefore, a structure of human SQLE has been long awaited.

## Shaping up SQLE

The architecture of the catalytic domain of human SQLE is finally unveiled by Padyana and coworkers^10^. They determined the crystal structures of the FAD-bound human SQLE alone and with two potent inhibitors NB-598 and Cmpd-4”. Despite interspersed primary sequence, the N-terminal-truncated protein contains three distinct domains, the FAD-binding domain, the substrate-binding domain, and a C-terminal helical membrane-binding domain (Fig. 2b). The two inhibitor-bound structures are very similar, with the inhibitors buried deep within an elongated hydrophobic tunnel that has an opening next to the flavin group. The unliganded structure exhibits slight conformational shifts in the substrate-binding domain compared to the inhibitor-bound ones. In particular, a conserved Y195 residue, which is hydrogen-bonded with the inhibitors in the substrate-binding domain, swings away from the hydrogen bond with residue Q168 in the FAD-binding domain. Mutating Y195 almost completely abolished catalytic activity, supporting the functional significance of the interaction between Y195-Q168.

The inhibitor-binding tunnel is likely also the squalene binding site and the authors present a squalene bound SQLE model based on docking experiments. Interestingly, despite the structural implication, kinetic analyses suggest non-competitive inhibition by NB-598 and Cmpd-4”, which the authors attributed to a slow and tight binding mode of the inhibitors. The structures also offer insight into the mechanism of action for stereo- and regio-specific catalysis by SQLE.

## The missing cholesterol-regulatory domain

As previously reported^6^, the hydrophobic N-terminal domain causes problems when trying to purify the full-length form of mammalian SQLE. The crystallized construct lacks the first 117 amino acids, representing a fifth of the 574 amino acid enzyme^10^. Importantly, this missing region includes the cholesterol-regulatory domain. Cholesterol accelerates degradation of SQLE, via the N-terminal 100 amino acids, an example of end-product inhibition at the post-translational level. This domain is pinned into the endoplasmic membrane via a re-entrant loop followed by a 12 amino acid stretch comprising a spring-loaded amphipathic helix that is ejected from the membrane with excess cholesterol to initiate proteasomal degradation of SQLE^11^. Therefore, while the structure of the important regulatory domain remains unknown, biochemical approaches have helped to elucidate its membrane topology and function.

## SQLE strikes again

SQLE can act again to introduce a second epoxide to produce 2,3(*S*),22(*S*),23-dioxidosqualene^6^ (Fig. 1). This second epoxide is introduced on the corresponding position as the first on the other end of the isoprenoid, indicating that 2,3(*S*)-oxidosqualene can be flipped and reinserted into the substrate binding pocket, hydrocarbon head-first and that the same regio- and stereo-specificity applies. The significance of this second epoxidation is that it represents the entry-point for diverting the product 2,3(*S*),22(*S*),23-dioxidosqualene into a shunt in the biosynthesis pathway. This shunt pathway is most active when SQLE activity is high and the activity of the subsequent enzyme lanosterol synthase is low. The final product 24(*S*),25-epoxycholesterol appears to serve as a measure of cholesterol synthesis and to protect against surges in the production of this potentially cytotoxic molecule. In addition, endogenous 24(*S*),25-epoxycholesterol is a natural ligand for the liver X receptors which induce expression of cholesterol efflux-related genes as well as promoting dopaminergic neurogenesis^12, 13^.

## Unanswered questions and future directions

Given the authors’ pharmaceutical background, their focus on inhibitors is understandable. From a more basic biology viewpoint, more structural information may be required to elucidate the actions of SQLE on its native substrates squalene and 2,3*(S)*-oxidosqualene. However, no well-diffracting crystals of the SQLE•FAD complex with either of the substrates could be obtained. Squalene is a large hydrophobic molecule without a polar group to anchor it to the aqueous interface, and so may reside in the midplane of the lipid bilayer^14^. But how is squalene extracted from the membrane to be acted on by SQLE? Presumably, supernatant protein factor (perhaps in concert with anionic phospholipids) transfers squalene from the membrane midplane to the active site of SQLE, but it is not known how the catalytic domain of SQLE associates with the membranes of the endoplasmic reticulum. The hydrophobic regions at the C-terminus are probably membrane associated perhaps via two putative transmembrane domains (Fig. 2) and future studies are needed to investigate the interactions of SQLE, supernatant protein factor and the membrane.

Terbinafine, a common antifungal agent, has been proposed as a new treatment strategy for human cancers^4^. Terbinafine shares the tertiary amine motif of the two inhibitors tested but has a naphthalene moiety which is bulkier than the corresponding moieties in either NB-598 or Cmpd-4”. Accordingly, Padyana and co-workers^10^ found terbinafine to be a far less potent inhibitor, with an IC_50_ of 7.7 μM compared to 63 and 69 nM for NB-598 or Cmpd-4”, respectively. The idea that terbinafine is a weak partial inhibitor against human SQLE fits neatly with species differences in the residues lining the binding pocket. Moreover, the crystal structure of the human enzyme proved useful for rationalizing why mutations of residues lining the binding pocket render certain fungal strains resistant to terbinafine treatment. Therefore, the SQLE structure will stimulate the development of better inhibitors that are more potent and selective against either the human or fungal versions of this enzyme.

Mahoney et al.^5^ investigated NB-598 sensitivity in numerous cancer cell-lines of neuroendocrine origin, including from small cell lung cancers. Using multiple approaches, they concluded that NB-598 sensitivity was due to toxicity caused by squalene accumulation rather than to insufficient cholesterol being synthesized. The enhanced toxicity relied on lipid droplets serving as storage for excess squalene, since inhibition of lipid droplet formation made even insensitive cells highly vulnerable to NB-598. The link between SQLE, squalene and lipid droplets clearly warrants further research, particularly in an oncogenic context.

Therefore, the crystal structure of the catalytic domain of human SQLE is certainly a boon to the growing throng of researchers interested in this fascinating enzyme vital in human health and disease. Indeed, the accompanying chemical biology study provides more compelling evidence that SQLE has a prominent role in certain cancers.
